# Quantifying the relative effects of environmental and direct transmission of norovirus

**DOI:** 10.1098/rsos.170602

**Published:** 2018-03-07

**Authors:** S. Towers, J. Chen, C. Cruz, J. Melendez, J. Rodriguez, A. Salinas, F. Yu, Y. Kang

**Affiliations:** Simon A. Levin Mathematical, Computational and Modeling Sciences Center, Arizona State University, Tempe, AZ, USA

**Keywords:** norovirus, mathematical model, basic reproduction number, epidemiology

## Abstract

Norovirus is a common cause of outbreaks of acute gastroenteritis in health- and child-care settings, with serial outbreaks also frequently observed aboard cruise ships. The relative contributions of environmental and direct person-to-person transmission of norovirus have hitherto not been quantified. We employ a novel mathematical model of norovirus transmission, and fit the model to daily incidence data from a major norovirus outbreak on a cruise ship, and examine the relative efficacy of potential control strategies aimed at reducing environmental and/or direct transmission. The reproduction number for environmental and direct transmission combined is $R_{0}^{\mathrm{tot}}=7.2$ [6.1,9.5], and of environmental transmission alone is $R_{0}^{\mathrm{environ}}=1.6$ [0.9,2.6]. Direct transmission is overwhelmingly due to passenger-to-passenger contacts, but crew can act as a reservoir of infection from cruise to cruise. This is the first quantification of the relative roles of environmental and direct transmission of norovirus. While environmental transmission has the potential to maintain a sustained series of outbreaks aboard a cruise ship in the absence of strict sanitation practices, direct transmission dominates. We find that intensive promotion of good hand washing practices may prevent outbreaks. Isolation of ill passengers and cleaning are beneficial, but appear to be less efficacious at outbreak control.

## 1. Background

Noroviruses are a group of non-enveloped, single-stranded RNA viruses [1,2], and are a highly infectious causal agent of sporadic and epidemic gastroenteritis [3,4]; they are the most common cause of gastroenteritis, food-borne disease and community acquired diarrhoeal disease across all ages in the USA [1,5,6]. Noroviruses are spread primarily through faecal–oral transmission, exposure to vomit, consumption of food or drink that has been contaminated, contact with contaminated surfaces or direct contact with those infected [7,8]. Each year in the USA, noroviruses cause, on average, 19–21 million cases of acute gastroenteritis (AG) [9], leading to 1.7–1.9 million outpatient visits and 400 000 emergency department visits, 56 000–71 000 hospitalizations and 570–800 deaths, mostly among young children and the elderly [9]. The frequent occurrence of outbreaks has resulted in the substantial economic burden of $500 million for norovirus-associated hospitalizations in the USA [10].

One of the key challenges that noroviruses pose is short-lived immunity with limited cross protection between strains, enabling multiple potential infections with the viruses through the lifetime of the host [3,4,11]. Additionally, the environmental durability of norovirus leads to persistence of the pathogen in clinical settings, and other closed or semi-closed environments such as daycare centres, schools and cruise ships, thus complicating complete disinfection and allowing for recurrent outbreaks [5,12,13]. There are currently no commercially available vaccines or specific treatments (other than palliative treatment) for noroviruses, leading to sanitation, personal hygiene practices, and quarantine or isolation as being the only potential means of control of the spread of the disease [14].

Cruise ships have been frequent settings of norovirus outbreaks [13,15–17], with the potential for significant negative economic impact. With approximately 25 million passengers annually across the world and growing, almost one million full-time equivalent jobs with $38 billion in wage and salaries and over $100 billion economic impact worldwide, the cruise industry has an influence on the lives of people in terms of recreation and employment.^1^ Unfortunately, the environment on cruise ships has the ingredients of a ‘perfect storm’ for outbreaks, with common food and drink, shared spaces for most activities and a semi-closed environment [5,16]. The economic disincentive for passengers and crew to report illness may also play a role in complicating control of outbreaks [5]. Even with good performance on environmental health sanitation inspections, it has been noted that outbreaks of gastroenteritis per 1000 cruises have been increasing in time [18], indicating that sanitation is not the only factor that must be considered in outbreak control.

Statistical analyses based on survey questionnaires have examined the relationships between certain behaviours and norovirus outbreaks. For example, a systematic review of the literature associated with 127 past norovirus outbreaks on cruise ships showed that preventative measures, such as food hygiene or the promotion of preventative procedures, could potentially affect outcomes [16]. Another survey found that passengers with a lackadaisical attitude towards hand hygiene were more likely to be infected with norovirus during an outbreak [13].

Beyond statistical analyses, however, mathematical models that describe the dynamics of the spread of a pathogen can yield insights into the relative efficacy of potential control measures [19]. There have been several previous works that explore the dynamics of the spread of norovirus:

— Vanderpas *et al.* [20] constructed a simple deterministic compartment model to analyse a norovirus outbreak in long-term care facilities in a closed population [20], which highlighted the need for reinforced infection control measures.
— Simmons *et al.* [11] constructed an age-structured deterministic transmission model to examine the duration of immunity after infection, and the age-dependence of transmission [11].
— Zelner and co-workers [21] examined a compartmental model that incorporated temporal changes in infectiousness, to examine the implications for control of transmission in a household setting.
— Bartsch *et al.* [22] constructed an agent-based model to simulate the spread of norovirus within and between 29 acute care hospitals and five long-term care facilities [22], and concluded that control was better achieved when hospitals acted cooperatively to track outbreaks.
— Lopman *et al.* [23] developed a dynamic transmission model of norovirus infection, disease and immunity to gain an understanding of the apparent high prevalence of asymptomatic infection [23].
— Assab & Temmime [24] used a stochastic model to examine the effects of hand washing and isolation on the spread of norovirus within nursing homes.
— Lee *et al.* [25] developed simulation models to determine the potential cost-savings from the hospital perspective of implementing various norovirus outbreak control interventions [25].

However, until now, no norovirus model has examined the relative contributions of environmental and person-to-person transmission to the overall transmissibility of the virus within a population. In addition, no model has incorporated the unique temporal dynamics of outbreaks on cruise ships, which can exhibit multiple waves from cruise to cruise as the passenger population refreshes with each new cruise, while the crew population remains the same, potentially acting as a reservoir of infection. Incorporation of these dynamics into a mathematical model of norovirus transmission can aid in assessing the relative efficacy of control strategies aimed at sanitation, hygiene, quarantine and/or isolation of sick patients, as the temporal dynamics of incidence in the cruise passengers and crew can help elucidate the relative contributions of the different modes of transmission.

The main objectives of our analysis are threefold:

1. To explore the dynamical effects of both environmental and direct transmission of norovirus on cruise ships by constructing a realistic mathematical model.
2. Optimize the model parameters to outbreak data.
3. Use the model to provide insights to help inform effective control strategies in a cruise ship setting.

In the following sections, we will describe our data and mathematical model, followed by a presentation of results and discussion.

## 2. Material and methods

### 2.1. Data

**Figure 1.**
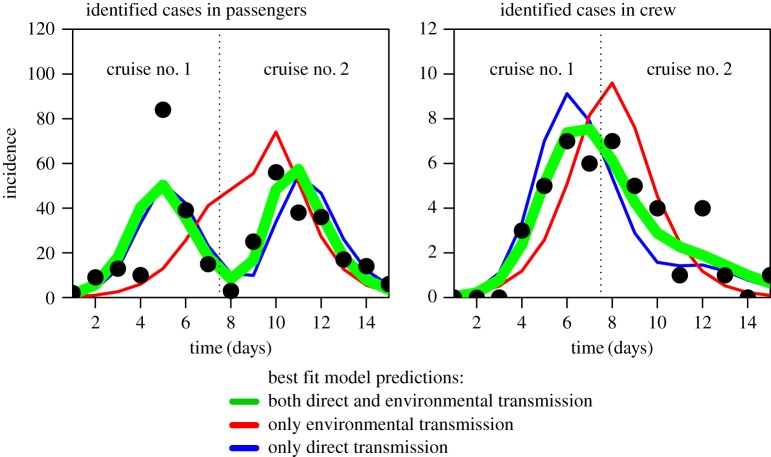
Time series of reported acute gastroenteritis cases, by date of symptom onset, for passengers and crew aboard two consecutive cruises aboard a cruise ship during late 2002, from [26]. Overlaid is the best-fit model of equations (2.2) that includes both direct and environmental transmission (green), along with the best-fit model assuming only environmental transmission (red), and only direct transmission (blue).

Data for these studies were the time series of the daily number of identified cases of AG among 2300–2400 passengers and 999 crew members during a six-week period aboard a cruise ship in late 2002 [26]. Weekly cruises took place, with new passengers at each cruise, but with the crew members remaining the same from cruise to cruise. The first two cruises recorded the largest number of AG cases, which laboratory testing of stool samples revealed to be primarily caused by a single strain of norovirus. The ship was taken out of service for a week after the first two cruises for thorough sanitation, but AG cases continued (although at a decreased level) for several subsequent cruises. Laboratory analysis of stool samples from identified AG cases in the later cruises revealed that six different norovirus strains were involved. We confine this analysis to the examination of the time-series data from the first two cruises, which involved the single strain of norovirus, to avoid the complications of taking into account cross-immunity between strains. The time-series data for passengers and crew members are shown in figure 1.

From a survey study of the identified cases, the investigators of the outbreak concluded that the initial index case(s) may have been due to potential food contamination, but that secondary cases were likely due to person-to-person infection (with the crew potentially acting as reservoir of infection from cruise to cruise), and potential environmental fomite transmission [26].

### 2.2. Mathematical model

In this study, we employ a deterministic susceptible, exposed, infected and recovered type of mathematical model to simulate the transmission dynamics of norovirus on a cruise ship. Similar models have been used in the past studies of norovirus transmission [20,23], and have also been used to examine a wide array of diseases, including influenza, measles and Ebola, to name but a few [27]. We modify the model to include environmental contamination, via an additional model compartment, *W*, similar to some cholera models [28,29].

Additionally, we incorporate the potentially differing direct transmission dynamics within, and between, the two primary sub-populations on a cruise ship: the crew and passengers. In the model, the crew members contact (and can be infected upon exposure) infectious crew members or passengers, whereupon they move to the exposed, but not yet infectious, compartment. After a period of time, 1/*κ*, they proceed to the infectious compartment. They recover with immunity after 1/*γ* days. Similar to the crew, passengers also contact crew and other passengers, with potentially differing rates compared to the contacts the crew make, and can be infected by infectious individuals in both groups.

Because a cruise season is much shorter than the typical duration of immunity upon recovery from norovirus, we ignore waning immunity in the model. We also ignore births and deaths in the model (other than the influx of new passengers from cruise to cruise).

The inclusion of both environmental and direct transmission is novel for modelling studies of the transmission of norovirus in any setting, and the inclusion of the dynamics of interaction between crew and passengers is novel for the study of norovirus transmission aboard cruise ships.

Past studies have shown that a significant fraction of norovirus cases are asymptomatic [7,30,31]. We, thus, include in the model an infected and asymptomatic class for both passengers and crew. The infected asymptomatic individuals can transmit infection, with a discount-on-transmission parameter, *σ*, relative to infected symptomatic individuals, with 0≤*σ*≤1.

The differential equations describing these dynamics are as follows:

2.1 $$\begin{array}{rl} \frac{dS_{i}}{dt} & =-\eta_{W}WS_{i}-\frac{S_{i}\sum_{j}B_{ij}I_{j}}{N_{j}}-\frac{S_{i}\sigma \sum_{j}B_{ij}I_{j}^{\mathrm{asymp}}}{N_{j}}\\ \frac{dE_{i}}{dt} & =+\eta_{W}WS_{i}+\frac{S_{i}\sum_{j}B_{ij}I_{j}}{N_{j}}+\frac{S_{i}\sigma \sum_{j}B_{ij}I_{j}^{\mathrm{asymp}}}{N_{j}}-\kappa E_{i}\\ \frac{dI_{i}}{dt} & =+\left(1-f_{\mathrm{asymp}}\right)\kappa E_{i}-\gamma I_{i}\\ \frac{dI_{i}^{\mathrm{asymp}}}{dt} & =+f_{\mathrm{asymp}}\kappa E_{i}-\gamma I_{i}^{\mathrm{asymp}}\\ \frac{dR_{i}}{dt} & =+\gamma I_{i}+\gamma I_{i}^{\mathrm{asymp}}\\ \;\text{and}\;\frac{dW}{dt} & =+\alpha \sum I_{i}+\sigma \alpha \sum I_{i}^{\mathrm{asymp}}-\xi W, \end{array}\}$$

where the sub-population indices, *i* and *j*, refer to ‘passengers’ when *i*,*j*=1, and ‘crew’ when *i*,*j*=2. The parameter *B*_*ij*_ is the contact rate, sufficient to transmit infection, between individuals in sub-population *i* and those in sub-population *j*. The parameter *η*_*W*_ is the rate at which the population contacts the environment, and *α* and *ξ* are the excretion and decay rates of the pathogen into, and out of, the environment, respectively. We also have the population size $N=N_{1}+N_{2}=S_{1}+E_{1}+I_{1}+I_{1}^{\mathrm{asymp}}+R_{1}+S_{2}+E_{2}+I_{2}+I_{2}^{\mathrm{asymp}}+R_{2}$.

Because the population is closed, the amount of time passengers spend with crew must equal the amount of time crew spends with passengers. Thus under the assumption that the probability of transmission upon contact is the same for both groups, the transmission matrix must satisfy reciprocity [32], which means that *N*_1_*B*_12_=*N*_2_*B*_21_.

Following [28], we re-scale the environmental compartment of equations (2.1), such that $W_{\mathrm{new}}\to \frac{\xi }{\alpha N}W_{\mathrm{old}}$. This yields

2.2 $$\begin{array}{rl} \frac{dS_{i}}{dt} & =-\eta_{W}WS_{i}-\frac{S_{i}\sum_{j}B_{ij}I_{j}}{N_{j}}-\frac{S_{i}\sigma \sum_{j}B_{ij}I_{j}^{\mathrm{asymp}}}{N_{j}}\\ \frac{dE_{i}}{dt} & =+\eta_{W}WS_{i}+\frac{S_{i}\sum_{j}B_{ij}I_{j}}{N_{j}}+\frac{S_{i}\sigma \sum_{j}B_{ij}I_{j}^{\mathrm{asymp}}}{N_{j}}-\kappa E_{i}\\ \frac{dI_{i}}{dt} & =+\left(1-f_{\mathrm{asymp}}\right)\kappa E_{i}-\gamma I_{i}\\ \frac{dI_{i}^{\mathrm{asymp}}}{dt} & =+f_{\mathrm{asymp}}\kappa E_{i}-\gamma I_{i}^{\mathrm{asymp}}\\ \frac{dR_{i}}{dt} & =+\gamma I_{i}+\gamma I_{i}^{\mathrm{asymp}}\\ \;\text{and}\;\frac{dW}{dt} & =+\xi \left(\frac{\sum \left(I_{i}+\sigma I_{i}^{\mathrm{asymp}}\right)}{N}-W\right), \end{array}\}$$

with scaled environmental transmission rate *β*_*W*_=*η*_*W*_*Nα*/*ξ*.

**Figure 2.**
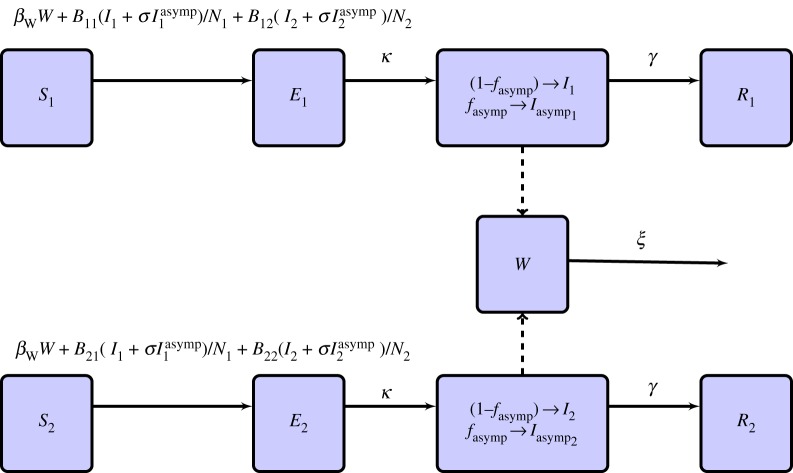
Compartmental flow diagram of the model described in equations (2.2). In the diagram, subscripts 1 and 2 refer to the passenger and crew sub-populations, respectively.

The compartmental diagram for the model of equations (2.2) is shown in figure 2, and a summary of the parameters of the model is given in table 1.

**Table 1.** Parameters of the model of equations (2.2). Quantities described below the dashed line are derivative of the parameters above the line.

parameter	description	value	reference
*N*_1_ (*N*_pass_)	number of passengers	2318	[26]
*N*_2_ (*N*_crew_)	number of crew	999	[26]
*B*_11_	rate passengers contact passengers	TBD	
*B*_12_	rate passengers contact crew	TBD	
*B*_22_	rate crew contact crew	TBD	
*B*_21_	rate crew contact passengers	*N*_1_*B*_11_/*N*_2_	[32]
*β*_*W*_	environmental transmission rate	TBD	
*f*_susc_	fraction of the population initially susceptible	TBD	
*σ*	relative transmissibility of asymptomatic individuals	TBD	
1/*κ*	incubation period	[1.1,1.2] days	[33]
1/*γ*	infectious period	1.17 [1.00,1.88] days	[34]
*ξ*	decay rate of norovirus in the environment	0.61 [0.38,0.84] days^−1^	[35]
*f*_asymp_	fraction of cases that are asymptomatic	0.32 [0.18,0.48]	[30,31]
*f*^passengers^_confirmed_	fraction symptomatic cases confirmed in passengers	TBD	
*f*^crew^_confirmed_	fraction symptomatic cases confirmed in crew	TBD	
$R_{0}^{\mathrm{tot}}$	basic reproduction number of direct and environmental transmission	TBD	
$R_{0}^{\mathrm{direct}}$	basic reproduction number of direct transmission alone	TBD	
$R_{0}^{\mathrm{environ}}$	basic reproduction number of environmental transmission alone	TBD	

The basic reproduction number of a disease is the average number of new infections produced by a single infectious individual during the course of their infection, in a completely susceptible population [19,36]. As described in electronic supplementary material, appendix A, using the next-generation matrix approach [36], we find that the reproduction number of the model of equations (2.2) is

2.3 $$R_{0}^{\mathrm{tot}}=q\frac{B_{11}+B_{22}+\beta_{W}+A}{2\gamma },$$

where

$$A=\sqrt{{\left(B_{11}-B_{22}+(f_{1}-f_{2})\beta_{W}\right)}^{2}+4*f_{1}*f_{2}\left(\frac{B_{12}}{f_{2}}+\beta_{W}\right)\;\left(\frac{B_{21}}{f_{1}}+\beta_{W}\right)}$$

and where *f*_*i*_ is the fraction of the population in sub-group *i*, and

2.4 $$q=(1-f_{\mathrm{asymp}})+\sigma f_{\mathrm{asymp}}.$$

In the absence of direct transmission (i.e. transmission occurs through the environment only), *B*_*ij*_=0 for all *i* and all *j*, and equation (2.3) simplifies to

2.5 $$R_{0}^{\mathrm{environ}}=\frac{q\beta_{W}}{\gamma }.$$

A cruise ship has regular emigration and immigration of passengers (but not the crew). We thus assume that the initial conditions at the beginning of the first cruise in the data are one infected symptomatic passenger introduced to a population of 999 crew and 2317 other passengers [26], where a fraction, *f*_susc_, of the crew and passengers are susceptible to catching norovirus, and a fraction (1−*f*_susc_) has been previously exposed at some time in the past, and are recovered and immune. When *f*_susc_<1, the reproduction number of the outbreak is scaled by $f_{\mathrm{susc}}R_{0}$, where $R_{0}$ is as in equations (2.3). At the beginning of the subsequent cruise, the passengers are replaced with a group of new passengers, a fraction, *f*_susc_, of whom are susceptible, while the crew population remains. Because of the limited duration of the cruises and the temporal variation in the refreshing of the passenger population with new groups of susceptible individuals, the model cannot be analytically analysed to estimate outbreak final size. Rather, we must rely on numerical simulations.

Not all symptomatic cases of norovirus aboard the ship were necessarily identified, and there is the historical evidence for low rates of cruise passengers with symptomatic gastroenteritis seeking healthcare aboard the ship; past survey studies of cruises, where outbreaks of norovirus have occurred, have shown that up to 70% of symptomatic passengers either delayed reporting to the infirmary, or did not report at all [13,37]. The reasons for the avoidance of healthcare were, in part, due to individuals feeling their symptoms were not serious enough to warrant treatment, and also due to a desire to avoid enforced isolation [13]. In addition, crew members on many ships can suffer economic disadvantages if they take time off due to illness; according to the Glassdoor employer review website (www.glassdoor.com, accessed April 2017), where employees can leave anonymous reviews of their employers, several major cruise lines are reported to be rather less than accommodating when it comes to paid sick leave for crew members. To estimate the fraction of symptomatic cases that are identified in crew and passengers (*f*^crew^_confirmed_ and *f*^passengers^_confirmed_, respectively), we thus divide the number of identified cases in the crew and passengers by the model estimates for the total number of symptomatic cases within each of the sub-populations.

With our model, the efficacy of reducing environmental transmission through cleaning can be examined (which results in an increase in the pathogen environmental decay rate, *ξ*, and a proportional decrease in *β*_*W*_). We also examine interventions aimed at immediate and complete isolation of some fraction, *f*_isol_, of infectious individuals; as described in electronic supplementary material, appendix A, and [38], this results in a relative reduction in $R_{0}^{\mathrm{tot}}$ of (1−*f*_isol_). We also examine interventions aimed at reducing the overall contacts sufficient to transmit infection in the population, due, for example, to personal hygiene practices designed to reduce the probability of transmission on contact either directly with another person, or with a contaminated environmental surface (i.e. proportional reductions in both *B*_*ij*_ and *β*_*W*_).

To examine how isolation of symptomatic individuals affects the reduction in final size of the outbreak relative to the observed baseline, we assume that some fraction of symptomatic and infectious individuals, *f*_isol_, are moved to completely effective isolation immediately upon showing symptoms. To examine how environmental cleaning affects the outbreak final size, we proportionately scale both the environmental transmission rate, *β*_*W*_, and the environmental decay rate, *ξ*, by a factor *ρ*, where 0≤*ρ*≤1. To examine the effect of hand washing, we proportionately scale the direct transmission rates, *B*_*ij*_, and the environmental transmission rate, *β*_*W*_, by a factor, *ρ*, where 0≤*ρ*≤1.

To assess the sensitivity of predicted efficacy of these intervention measures to our estimated uncertainties on the model parameters, we repeat the assessments with 1000 Monte Carlo iterations for each proposed intervention, randomly sampling the model parameters at each iteration from the probability distribution for the uncertainty on each model parameter (these parameters include *B*_*ij*_, *β*_*W*_, *f*_asymp_, *f*_susc_, *σ*, *f*_asymp_, *κ*, *γ* and *ξ*).

### 2.3. Statistical methods

In our analysis, we fit the model predictions of equations (2.2) for the daily number of symptomatic confirmed cases in the crew and passengers to the observed number of cases in the Isakbaeva *et al.* cruise outbreak data [26]. The fits estimate the transmission rate parameters *β*_*W*_, *B*_11_, *B*_12_ and *B*_22_ of the model shown in equations (2.2), using a negative binomial likelihood fit to account for over-dispersion in the data [39].

In addition, we fit for the initial fraction of susceptible individuals in the crew and passenger populations, *f*_susc_, and we also fit for the relative discount-on-transmission, *σ*, of asymptomatic individuals. A graphical Monte Carlo method was used in the optimization procedure [40].

We also fit for the incubation and infectious periods, 1/*κ* and 1/*γ*, respectively, with the likelihood modified to include the Bayesian prior probability distributions for these values, as obtained from previous studies:

— The incubation period of norovirus in a community setting has been estimated by some studies to be approximately 2 days [2,41], and by another meta-analysis study to have 95% confidence interval [1.1,1.2] days [33].
— There are few estimates of the infectious period of norovirus, although it is suspected it is longer than the duration of symptoms [21,42]. An analysis of a norovirus community outbreak in Sweden estimated that 1/*γ* is 1.17 days with 95% CI [1.00,1.88] [34].

To incorporate this prior information into our likelihood fit, we assume that the prior probability distribution for 1/*κ* is normal, with mean 1.15 days and standard deviation 0.1/1.96/2=0.03 days. We assume that the prior probability distribution for 1/*γ* is an asymmetric normal distribution, with mean *μ*=1.17 days, and standard deviation *δ*=(1.17−1)/1.96=0.09 days when 1/*γ*≤*μ*, and *δ*=(1.88−1.17)/1.96=0.36 days when 1/*γ*>*μ*. Given hypotheses for 1/*κ* and 1/*γ*, the likelihood is then modified by multiplying the likelihood by the probabilities calculated from these two distributions. Because there is some discrepancy in the prior estimates of 1/*κ*, with the estimate of 2 days from [2,41], and the 95% CI estimate of [1.1,1.2] days from [33], we cross-check the results of the analysis by repeating the fit, removing the prior-belief likelihood constraint on *κ*.

The rate of decay in viability of norovirus on environmental surfaces is poorly known, largely because norovirus cannot be grown in cell culture [14,35]. However, the feline calicivirus (FCV) has been used by several investigators as an acceptable surrogate for norovirus in inactivation studies; Mattison *et al.* [35] examined the inactivation of FCV in food, and on metal at various temperatures [35]. From the time-series data presented in Mattison *et al.* [35] for FCV on metal surfaces at room temperature, we estimate that the exponential rate of decline of the virus is 0.607±0.117 *days*^−1^. In the fit of model equations (2.2) to the cruise outbreak data, we thus also fit for the rate of decay of the virus in the environment, *ξ*, with the likelihood modified with the normal prior probability distribution for *ξ*, with mean 0.607 *days*^−1^ and standard deviation 0.117 *days*^−1^.

We also fit for the fraction of infections that are asymptomatic, *f*_asymp_. Two separate analyses have estimated that the asymptomatic infection fraction in experimental volunteer infection studies, and community outbreak settings, is 32% [30,31]. In the fit of model equations (2.2) to the cruise outbreak data, we thus also fit for the asymptomatic fraction, *f*_asymp_, with the likelihood modified with the normal prior probability distribution for *f*_asymp_ based on the prior studies [30,31], with mean 0.34 and standard deviation 0.08.

## 3. Results

### 3.1. Model optimization

In table 2, we show the results of the optimization of the model parameters of equations (2.2) to the cruise outbreak data of Isakbaeva *et al.* [26]. In figure 1, we show the best-fit model of equations (2.2) overlaid on the data. The best-fit total reproduction number of the outbreak is $R_{0}^{\mathrm{tot}}=7.2$ with 95% CI [6.1,9.5]. The direct and environmental transmission components of the reproduction number are $R_{0}^{\mathrm{direct}}=5.9$ with 95% CI [4.7,8.1] and $R_{0}^{\mathrm{environ}}=1.6$ with 95% CI [0.9,2.6], respectively. As seen in table 2, the passenger-to-passenger contacts dominate the direct transmission; however, there is significant contact between passengers and crew.

**Table 2.** Results of the optimization of the model parameters of equations (2.2) to the cruise outbreak data of Isakbaeva *et al.* [26]. The table values below the dashed line are derivative of the model parameters above the dashed line. The best-fit model overlaid on the data is shown in figure 1.

*B*_11_	49 [28.8,60.6] days^−1^
*B*_12_	1.98 [0,5.38] days^−1^
*B*_22_	2.87 [0,18.89] days^−1^
*B*_21_	4.6 [0,12.48] days^−1^
(*B*_21_+*B*_22_)/(*B*_11_+*B*_12_)	0.15 [0.02,0.44]
*β*_*W*_	13.31 [4.45,19.32] days^−1^
*f*_susc_	0.12 [0.11,0.27]
*σ*	[0,1]
1/*κ*	1.15 [1.12,1.18] days
1/*γ*	1.15 [1.05,1.57] days
*ξ*	0.6 [0.39,0.83] days^−1^
*f*_asymp_	0.31 [0.22,0.41]
$R_{0}^{\mathrm{tot}}$	7.2 [6.1,9.5]
$R_{0}^{\mathrm{direct}}$	5.9 [4.7,8.1]
$R_{0}^{\mathrm{environ}}$	1.6 [0.89,2.61]
*f*^passengers^_confirmed_	0.99 [0.43,1.00]
*f*^crew^_confirmed_	0.57 [0.25,0.60]

Also overlaid in figure 1 are the best-fit models assuming no environmental transmission, and no direct transmission. The overall best-fit model to the temporal patterns of incidence in the crew and passengers is the model that includes both environmental and direct transmission.

The fraction of the population found to be susceptible prior to the outbreak is *f*_susc_=0.12 [0.11,0.27]. The fraction of infected cases that were found to be asymptomatic is *f*_asymp_=0.31 [0.22,0.41]. The fit had no sensitivity to the asymptomatic discount-on-transmission parameter, *σ*, returning a 95% CI [0,1].

The cross-check of the analysis by fitting the incubation period, 1/*κ*, without a prior-belief constraint on the likelihood yields an estimate of 1/*κ*=1.54 [0.85,2.35] days. This is statistically consistent with the previous estimate of 1/*κ* of [1.1,1.2] days that was obtained by Sukhrie *et al*. [33]. The previous estimate of 1/*κ* being approximately 2 days, obtained by [2,41], is also consistent with our results, but at the high end of the range of our 95% CI.

The estimated fraction of symptomatic cases in passengers and crew that were confirmed is *f*^passenger^_confirmed_=0.99 [0.43,1.00] and *f*^crew^_confirmed_=0.57 [0.25,0.60], respectively.

### 3.2. Evaluation of potential control measures

**Figure 3.**
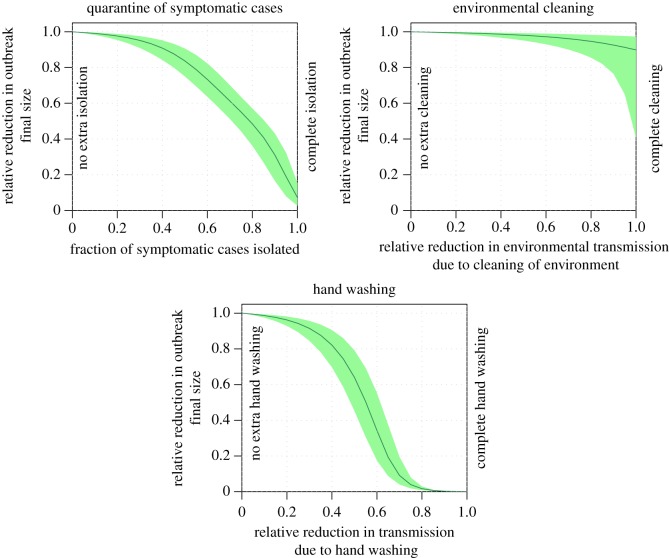
Effect of potential control strategies on outbreak final size, relative to the observed baseline. The shaded area represents the 95% CI on the estimates, due to the uncertainties on the model parameter estimates, as seen in table 2.

The results of the evaluation of control measures involving isolation of symptomatic individuals, environmental cleaning and hand washing are shown in figure 3. The shaded areas in figure 3 indicate the 95% CI on the estimated impact of the intervention measures, as obtained from the analysis of the sensitivity of the estimates to the uncertainties in the fitted model parameters. Note that our fits were found to not be sensitive to the asymptomatic discount-on-transmission parameter, *σ*, and thus the 95% CI on the assessment of the intervention measurements includes the uncertainty arising from values of *σ* between 0 and 1. Our model predicts that complete isolation of all symptomatic individuals would result in a relative reduction of 85% to 97% (95% CI) of the outbreak final size. Complete environmental cleaning would result in a final size relative reduction of 3% to 59% (95% CI), and completely efficacious and rigorously applied hand washing practices has the potential to reduce the final size by 100%.

## 4. Discussion

Our estimate of the overall reproduction number for the cruise ship outbreak is $R_{0}^{\mathrm{tot}}=7.2$ with 95% CI [6.1,9.5]. There have been few past estimates of the reproduction number of norovirus outbreaks; examination of norovirus outbreaks in hospitals and long-term care facilities has estimated the reproduction number to be between 1.1 and 3.4 [20,31], while examination of an outbreak at a large boy scout jamboree estimated the reproduction number to be $R_{0}=7.26$ with 95% CI [5.26,9.25] [43]. The larger reproduction number at the jamboree was likely due to the more active mixing of the population, unlike a hospital or long-term care facility where residents are largely bed-ridden. Our estimated reproduction number is in agreement with the reproduction number estimate of the jamboree, likely reflecting the similar mixing patterns in the populations being studied, with communal dining and many shared group activities.

It needs to be pointed out here that our model expression for the reproduction number in equations (2.2) is directly proportional to the infectious period 1/*γ*. The value of 1/*γ* for norovirus is poorly studied in the literature; in our analysis, we used values of 1/*γ* from prior observations of the course of illness in a community outbreak in Sweden, which estimated the infectious period to be between [1.00,1.88] days, with 95% confidence [34]. If, at a later date, a different estimate of 1/*γ* is obtained, the reproduction number estimates of our analysis merely need to be re-scaled by the new value.

We find that the fraction of people on the cruise initially susceptible to the circulating strain of norovirus was *f*_susc_=0.12 [0.11,0.27]. This is in agreement with studies that have found that the fraction of the older child and adult population with prior norovirus exposure, (1−*f*_susc_), appears to be approximately 80% to 90% [3,30,44,45].

Our analysis finds that the fraction of asymptomatic infection is *f*_asymp_=0.31 [0.22,0.41]. This relatively high asymptomatic fraction poses problems for control measures that isolate infectious passengers relying on symptomatic presentation alone. In addition, the situation is further complicated by the relatively low level of health-seeking behaviour by crew members; we estimate that only 57% [25%,60%] of symptomatic crew were identified as cases in the outbreak (*f*^crew^_confirmed_), likely in part due to the fear of lost wages should they report ill and be isolated. This is particularly problematic for outbreak control, because our analysis finds that crew are an important reservoir of infection from cruise to cruise. The health-seeking behaviour appears to be somewhat better in passengers aboard the cruise, with an estimate of between [43%,100%] 95% CI symptomatic cases being identified, but there is still the potential that many symptomatic passengers were not identified by the ship’s infirmary.

It should also be noted that our model assumes that ‘isolation’ consists of infectious and symptomatic individuals being isolated from other people in the population immediately upon becoming symptomatic. In reality, some time likely passes between onset of symptoms and isolation, and during that time, the individual can spread the infection to others. Our model thus represents the best case scenario for isolation practices. We, thus, find that isolation of symptomatic individuals, while beneficial in reducing the size of an outbreak, is unlikely to control an outbreak alone, as seen in figure 3.

The 95% CI on the reproduction number for environmental transmission, $R_{0}^{\mathrm{environ}}$, was found to be [0.89,2.61], and it is thus likely possible to achieve a sustained outbreak without direct transmission. Indeed, such sustained transmission due to fomite contact alone has been noted among consecutive groups of different people renting a houseboat, where there was no common population or direct contact between the consecutive groups [46]. Environmental cleaning thus has a role to play in the control of sustained outbreaks. However, it is worth noting that the cruise ship on which the outbreak occurred, like all cruise ships, had to pass a rigorous sanitation inspection [17], and thus it was already a quite clean environment. It only takes a few norovirus particles to transmit infection [47], and the pathogen is notoriously difficult to kill on surfaces [48]. It has been shown that wiping surfaces with common detergents served more to simply spread the pathogen around, rather than killing it [49]. Additionally, we find that direct transmission appears to be the dominant factor in outbreak size, and we find that even the most stringent cleaning that eliminated all the virus from the environment would result in a relative reduction in outbreak size over the two cruises of between 3% and 59% (95% CI).

Both the direct and environmental transmission can be substantially reduced through personal hygiene measures, such as hand washing, that reduce the probability of transmission upon contact (thus proportionally reducing both $R_{0}^{\mathrm{direct}}$ and $R_{0}^{\mathrm{environ}}$). Our analysis thus indicates that aggressive educational campaigns aimed at improved hand washing practices would potentially be most efficacious in reducing the morbidity burden, and effective, widespread hand washing can entirely prevent a potential outbreak. Indeed, the analysis of a norovirus outbreak at a boy scout jamboree found that the implementation of rigorous hand washing protocols reduced the reproduction number by 85% [43]. A survey analysis of a past norovirus outbreak aboard a cruise ship also found that lackadaisical attitude towards hand hygiene was one of the dominant risk factors affecting the probability of infection during the outbreak [37].

However, unlike children at a jamboree, who can be forced by adult authorities to wash their hands before eating, overcoming long-standing poor hygiene habits among some adults on a cruise ship can be a challenge [50]. Informational signs in bathrooms, and at entrances to ship eating areas, promoting the importance of proper hand washing might perhaps be useful, particularly if the signs stress the probable loss of quality vacation time for passengers who fall ill, while also pointing out that studies have shown that passengers who do not wash their hands before eating are much more likely to fall ill.

The model we employ in this analysis assumes homogeneous mixing of the sub-populations; that is to say, for example, that there are no preferential contacts between particular passengers, or between particular passengers and particular crew. This is unlikely to be completely true due to family or friend groupings, but the shared dining and many shared group activities of people aboard a cruise ship make the assumption of homogeneous mixing much more applicable in that environment than in most other environments within which humans typically interact. Including networked heterogeneities of contacts can affect the estimate of the reproduction number of the outbreak, and this must be kept in mind when comparing the results of this analysis with results of other analyses that rely on other means to assess the estimate of the reproduction number (by, for instance, using contact tracing).

## 5. Conclusion

We have presented a novel mathematical model for norovirus disease transmission aboard a cruise ship that includes both direct and environmental transmission. This is not the first model for a disease to include environmental transmission; for example, some cholera models include both direct and environmental transmission [28,51]. However, to the best of our knowledge, this is the first time that such a model has been used to quantify the relative contribution of direct and environmental transmission for norovirus disease. With the quantification of these relative contributions to transmission, the relative efficacy of potential control strategies aimed at either environmental sanitation, personal hygiene or isolation can be assessed.

We find that due to the relatively high asymptomatic fraction of norovirus infection, and relatively low rates of health-seeking behaviour, isolation of symptomatic passengers aboard a cruise ship is unlikely to be completely effectual for outbreak control. We also find that the rates of environmental transmission are high enough to likely result in sustained outbreaks, but that overall transmission is dominated by direct person-to-person contact. Thus, environmental cleaning is likely to have little impact on the final size of outbreaks on cruise ships. These findings are supported by past qualitative observations that norovirus outbreaks aboard cruise ships are notoriously difficult to control [13,15–17].

We find that reduction in environmental and direct transmission is potentially best achieved with personal hygiene measures, such as rigorous hand washing, designed to reduce the probability of infection upon contact with a contaminated surface or infectious individual. Further empirical studies in a cruise ship setting aimed at assessment of the efficacy of hand washing promotion campaigns would be helpful to confirm these results.

## Supplementary Material

Appendix A

## Footnotes

1 State of the Industry: 2017 Cruise Industry Outlook. Cruise Line International Associated; 2017.

## Data accessibility

All data and analysis scripts associated with this analysis can be downloaded from https://github.com/smtowers/norovirus.

## References

[1] Hall AJ, Vinjé J, Lopman B, Park GW, Yen C, Gregoricus N, Parashar U 2011 Updated norovirus outbreak management and disease prevention guidelines. *MMWR Recomm. Rep.* 60, 1–18. 21368741

[2] Weinstein RA, Said MA, Perl TM, Sears CL 2008 Gastrointestinal flu: norovirus in health care and long-term care facilities. *Clin. Infect. Dis.* 47, 1202–1208. (doi:10.1086/592299) 18808354 10.1086/592299

[3] Patel MM, Hall AJ, Vinjé J, Parashar UD 2009 Noroviruses: a comprehensive review. *J. Clin. Virol.* 44, 1–8. (doi:10.1016/j.jcv.2008.10.009) 19084472 10.1016/j.jcv.2008.10.009

[4] Glass RI, Parashar UD, Estes MK 2009 Norovirus gastroenteritis. *New England J. Med.* 361, 1776–1785. (doi:10.1056/NEJMra0804575) 19864676 10.1056/NEJMra0804575 PMC3880795

[5] Robilotti E, Deresinski S, Pinsky BA 2015 Norovirus. *Clin. Microbiol. Rev.* 28, 134–164. (doi:10.1128/CMR.00075-14) 25567225 10.1128/CMR.00075-14 PMC4284304

[6] Kaplan JE, Feldman R, Campbell DS, Lookabaugh C, Gary GW 1982 The frequency of a Norwalk-like pattern of illness in outbreaks of acute gastroenteritis. *Am. J. Public Health* 72, 1329–1332. (doi:10.2105/AJPH.72.12.1329) 6291414 10.2105/ajph.72.12.1329 PMC1650540

[7] Lopman B, Gastañaduy P, Park GW, Hall AJ, Parashar UD, Vinjé J 2012 Environmental transmission of norovirus gastroenteritis. *Curr. Opin. Virol.* 2, 96–102. (doi:10.1016/j.coviro.2011.11.005) 22440972 10.1016/j.coviro.2011.11.005

[8] Kampmeier S, Pettke A, Kossow A, Willems S, Mellmann A 2016 Norovirus infections in a tertiary care centre—individual cases do not necessarily lead to an outbreak. *J. Clin. Virol.* 84, 39–41. (doi:10.1016/j.jcv.2016.09.010) 27701033 10.1016/j.jcv.2016.09.010

[9] Hall AJ, Lopman BA, Payne DC, Patel MM, Gastañaduy PA, Vinjé J, Parashar UD 2013 Norovirus disease in the United States. *Emerg. Infect. Dis.* 19, 1198–1205. (doi:10.3201/eid1908.130465) 23876403 10.3201/eid1908.130465 PMC3739528

[10] Morris JG Jr, Hoffmann S, Batz B 2011 Ranking the risks: the 10 pathogen-food combinations with the greatest burden on public health. Emerging Pathogens Institute, University of Florida. See https://folio.iupui.edu/bitstream/handle/10244/1022/72267report.pdf?sequence=1.

[11] Simmons K, Gambhir M, Leon J, Lopman B 2013 Duration of immunity to norovirus gastroenteritis. *Emerg. Infect. Dis.* 19, 1260–1267. (doi:10.3201/eid1908.130472) 23876612 10.3201/eid1908.130472 PMC3739512

[12] Cheesbrough J, Green J, Gallimore C, Wright P, Brown D 2000 Widespread environmental contamination with Norwalk-like viruses (NLV) detected in a prolonged hotel outbreak of gastroenteritis. *Epidemiol. Infect.* 125, 93–98. (doi:10.1017/S095026889900432X) 11057964 10.1017/s095026889900432x PMC2869574

[13] Wikswo ME, Cortes J, Hall AJ, Vaughan G, Howard C, Gregoricus N, Cramer EH 2011 Disease transmission and passenger behaviors during a high morbidity Norovirus outbreak on a cruise ship, January 2009. *Clin. Infect. Dis.* 52, 1116–1122. (doi:10.1093/cid/cir144) 21429864 10.1093/cid/cir144

[14] Koo HL, Ajami N, Atmar RL, DuPont HL 2010 Noroviruses: the leading cause of gastroenteritis worldwide. *Discov. Med.* 10, 61–70. 20670600 PMC3150746

[15] Hadjichristodoulou C *et al.* 2011 Surveillance and control of communicable diseases related to passenger ships in Europe. *Int. Marit. Health* 62, 138–147. 21910118

[16] Bert F, Scaioli G, Gualano MR, Passi S, Specchia ML, Cadeddu C, Viglianchino C, Siliquini R 2014 Norovirus outbreaks on commercial cruise ships: a systematic review and new targets for the public health agenda. *Food Environ. Virol.* 6, 67–74. (doi:10.1007/s12560-014-9145-5) 24838574 10.1007/s12560-014-9145-5

[17] Lawrence DN 2004 Outbreaks of gastrointestinal diseases on cruise ships: lessons from three decades of progress. *Curr. Infect. Dis. Rep.* 6, 115–123. (doi:10.1007/s11908-996-0007-7) 15023273 10.1007/s11908-996-0007-7

[18] Cramer EH, Blanton CJ, Blanton LH, Vaughan GH, Bopp CA, Forney DL 2006 Epidemiology of gastroenteritis on cruise ships, 2001–2004. *Am. J. Prev. Med.* 30, 252–257. (doi:10.1016/j.amepre.2005.10.027) 16476642 10.1016/j.amepre.2005.10.027

[19] Hethcote HW 2000 The mathematics of infectious diseases. *SIAM Rev.* 42, 599–653. (doi:10.1137/S0036144500371907)

[20] Vanderpas J, Louis J, Reynders M, Mascart G, Vandenberg O 2009 Mathematical model for the control of nosocomial norovirus. *J. Hosp. Infect.* 71, 214–222. (doi:10.1016/j.jhin.2008.11.024) 19162373 10.1016/j.jhin.2008.11.024

[21] Milbrath M, Spicknall I, Zelner J, Moe C, Eisenberg J 2013 Heterogeneity in norovirus shedding duration affects community risk. *Epidemiol. Infect.* 141, 1572–1584. (doi:10.1017/S0950268813000496) 23507473 10.1017/S0950268813000496 PMC9155277

[22] Bartsch SM, Huang SS, Wong KF, Avery TR, Lee BY 2014 The spread and control of norovirus outbreaks among hospitals in a region: a simulation model. *Open Forum Infect. Dis.* 1, ofu030 (doi:10.1093/ofid/ofu030) 25734110 10.1093/ofid/ofu030 PMC4281820

[23] Lopman B, Simmons K, Gambhir M, Vinjé J, Parashar U 2014 Epidemiologic implications of asymptomatic reinfection: a mathematical modeling study of norovirus. *Am. J. Epidemiol.* 179, 507–512. (doi:10.1093/aje/kwt287) 24305574 10.1093/aje/kwt287

[24] Assab R, Temime L 2016 The role of hand hygiene in controlling norovirus spread in nursing homes. *BMC Infect. Dis.* 16, 395 (doi:10.1186/s12879-016-1702-0) 27507065 10.1186/s12879-016-1702-0 PMC4977681

[25] Lee BY, Wettstein ZS, McGlone SM, Bailey RR, Umscheid CA, Smith KJ, Muder RR 2011 Economic value of norovirus outbreak control measures in healthcare settings. *Clin. Microbiol. Infect.* 17, 640–646. (doi:10.1111/j.1469-0691.2010.03345.x) 20731684 10.1111/j.1469-0691.2010.03345.x PMC3005527

[26] Isakbaeva E *et al.* 2005 Norovirus transmission on cruise ship. *Emerg. Infect. Dis.* 11, 154–158. (doi:10.3201/eid1101.040434) 15705344 10.3201/eid1101.040434 PMC3294347

[27] Grassly NC, Fraser C 2008 Mathematical models of infectious disease transmission. *Nat. Rev. Microbiol.* 6, 477–487. (doi:10.1038/nrmicro1845) 18533288 10.1038/nrmicro1845 PMC7097581

[28] Tien JH, Earn DJ 2010 Multiple transmission pathways and disease dynamics in a waterborne pathogen model. *Bull. Math. Biol.* 72, 1506–1533. (doi:10.1007/s11538-010-9507-6) 20143271 10.1007/s11538-010-9507-6

[29] Chao DL, Longini IM Jr, Morris JG Jr 2013 Modeling cholera outbreaks. In *Cholera outbreaks*, pp. 195–209. Berlin, Germany: Springer (eds G Nair, Y. Takeda). Current Topics in Microbiology and Immunology, vol. 397 (doi:10.1007/82.2013.307)

[30] Graham DY, Jiang X, Tanaka T, Opekun AR, Madore HP, Estes MK 1994 Norwalk virus infection of volunteers: new insights based on improved assays. *J. Infect. Dis.* 170, 34–43. (doi:10.1093/infdis/170.1.34) 8014518 10.1093/infdis/170.1.34

[31] Sukhrie FHA, Teunis P, Vennema H, Copra C, Thijs Beersma MFC, Bogerman J, Koopmans M 2012 Nosocomial transmission of norovirus is mainly caused by symptomatic cases. *Clin. Infect. Dis.* 54, 931–937. (doi:10.1093/cid/cir971) 22291099 10.1093/cid/cir971

[32] Wallinga J, Teunis P, Kretzschmar M 2006 Using data on social contacts to estimate age-specific transmission parameters for respiratory-spread infectious agents. *Am. J. Epidemiol.* 164, 936–944. (doi:10.1093/aje/kwj317) 16968863 10.1093/aje/kwj317

[33] Lee RM, Lessler J, Lee RA, Rudolph KE, Reich NG, Perl TM, Cummings DA 2013 Incubation periods of viral gastroenteritis: a systematic review. *BMC Infect. Dis.* 13, 446 (doi:10.1186/1471-2334-13-446) 24066865 10.1186/1471-2334-13-446 PMC3849296

[34] Zelner JL, King AA, Moe CL, Eisenberg JN 2010 How infections propagate after point-source outbreaks: an analysis of secondary norovirus transmission. *Epidemiology* 21, 711–718. (doi:10.1097/EDE.0b013e3181e5463a) 20508526 10.1097/EDE.0b013e3181e5463a

[35] Mattison K, Karthikeyan K, Abebe M, Malik N, Sattar SA, Farber JM, Bidawid S 2007 Survival of calicivirus in foods and on surfaces: experiments with feline calicivirus as a surrogate for norovirus. *J. Food Prot.* 70, 500–503. (doi:10.4315/0362-028X-70.2.500) 17340890 10.4315/0362-028x-70.2.500

[36] Diekmann O, Heesterbeek J, Roberts M 2009 The construction of next-generation matrices for compartmental epidemic models. *J. R. Soc. Interface* 7, 873–885. (doi:10.1098/rsif.2009.0386) 19892718 10.1098/rsif.2009.0386 PMC2871801

[37] Neri AJ, Cramer EH, Vaughan GH, Vinjé J, Mainzer HM 2008 Passenger behaviors during norovirus outbreaks on cruise ships. *J. Travel Med.* 15, 172–176. (doi:10.1111/j.1708-8305.2008.00199.x) 18494694 10.1111/j.1708-8305.2008.00199.x

[38] Feng Z 2007 Final and peak epidemic sizes for SEIR models with quarantine and isolation. *Math. Biosci. Eng.: MBE* 4, 675–686. (doi:10.3934/mbe.2007.4.675) 17924718 10.3934/mbe.2007.4.675

[39] Lloyd-Smith JO 2007 Maximum likelihood estimation of the negative binomial dispersion parameter for highly overdispersed data, with applications to infectious diseases. *PLoS ONE* 2, e180 (doi:10.1371/journal.pone.0000180) 17299582 10.1371/journal.pone.0000180 PMC1791715

[40] Cowan G 1998 *Statistical data analysis*. Oxford, UK: Oxford University Press.

[41] Rockx B, de Wit M, Vennema H, Vinjé J, de Bruin E, van Duynhoven Y, Koopmans M 2002 Natural history of human calicivirus infection: a prospective cohort study. *Clin. Infect. Dis.* 35, 246–253. (doi:10.1086/341408) 12115089 10.1086/341408

[42] Atmar RL, Opekun AR, Gilger MA, Estes MK, Crawford SE, Neill FH, Graham DY 2008 Norwalk virus shedding after experimental human infection. *Emerg. Infect. Dis.* 14, 1553–1557. (doi:10.3201/eid1410.080117) 18826818 10.3201/eid1410.080117 PMC2609865

[43] Heijne JCM, Teunis P, Morroy G, Wijkmans C, Oostveen S, Duizer E, Kretzschmar M, Wallinga J 2009 Enhanced hygiene measures and norovirus transmission during an outbreak. *Emerg. Infect. Dis.* 15, 24–30. (doi:10.3201/eid1501.080299) 19116045 10.3201/1501.080299 PMC2660689

[44] Dai Y-c, Nie J, Zhang X-f, Li Z-f, Bai Y, Zeng Z-r, Yu S-y, Farkas T, Jiang X 2004 Seroprevalence of antibodies against noroviruses among students in a Chinese military medical university. *J. Clin. Microbiol.* 42, 4615–4619. (doi:10.1128/JCM.42.10.4615-4619.2004) 15472318 10.1128/JCM.42.10.4615-4619.2004 PMC522286

[45] Nurminen K, Blazevic V, Huhti L, Räsänen S, Koho T, Hytönen VP, Vesikari T 2011 Prevalence of norovirus GII-4 antibodies in Finnish children. *J. Med. Virol.* 83, 525–531. (doi:10.1002/jmv.21990) 21264875 10.1002/jmv.21990

[46] Jones EL, Kramer A, Gaither M, Gerba CP 2007 Role of fomite contamination during an outbreak of norovirus on houseboats. *Int. J. Environ. Health Res.* 17, 123–131. (doi:10.1080/09603120701219394) 17616868 10.1080/09603120701219394

[47] Dolin R 2007 Noroviruses–challenges to control. *New England J. Med.* 357, 1072–1073. (doi:10.1056/NEJMp078050) 17855667 10.1056/NEJMp078050

[48] Feliciano L, Li J, Lee J, Pascall MA 2012 Efficacies of sodium hypochlorite and quaternary ammonium sanitizers for reduction of norovirus and selected bacteria during ware-washing operations. *PLoS ONE* 7, e50273 (doi:10.1371/journal.pone.0050273) 23227163 10.1371/journal.pone.0050273 PMC3515596

[49] Barker J, Vipond I, Bloomfield S 2004 Effects of cleaning and disinfection in reducing the spread of Norovirus contamination via environmental surfaces. *J. Hosp. Infect.* 58, 42–49. (doi:10.1016/j.jhin.2004.04.021) 15350713 10.1016/j.jhin.2004.04.021

[50] Curtis VA, Danquah LO, Aunger RV 2009 Planned, motivated and habitual hygiene behaviour: an eleven country review. *Health Educ. Res.* 24, 655–673. (doi:10.1093/her/cyp002) 19286894 10.1093/her/cyp002 PMC2706491

[51] Tuite AR, Tien J, Eisenberg M, Earn DJ, Ma J, Fisman DN 2011 Cholera epidemic in Haiti, 2010: using a transmission model to explain spatial spread of disease and identify optimal control interventions. *Ann. Intern. Med.* 154, 593–601. (doi:10.7326/0003-4819-154-9-201105030-00334) 21383314 10.7326/0003-4819-154-9-201105030-00334

