# Supplementary material for: Quantifying the relative effects of environmental and direct transmission of norovirus
Source: R Soc Open Sci. 2018 Mar 7;5(3):170602. doi: 10.1098/rsos.170602 (PMC5882666; doi:10.1098/rsos.170602)
Supplement: Appendix A [file rsos170602supp1.pdf]

1 Appendix: Calculation of the reproduction number

1.1 Model with direct transmission alone

The ordinary differential equations for a Susceptible, Exposed, Infected, Recovered (SEIR) model with direct transmission within and between two interacting sub-populations, and both symptomatic and asymptomatic transmission, is

$$\begin{aligned}
\frac{dS_i}{dt} &= -S_i \sum_j B_{ij} I_j / N_j - S_i \sigma \sum_j B_{ij} I_j^{\text{asymp}} / N_j \\
\frac{dE_i}{dt} &= +S_i \sum_j B_{ij} I_j / N_j + S_i \sigma \sum_j B_{ij} I_j^{\text{asymp}} / N_j - \kappa E_i \\
\frac{dI_i}{dt} &= +(1 - f_{\text{asymp}}) \kappa E_i - \gamma I_i \\
\frac{dI_i^{\text{asymp}}}{dt} &= +f_{\text{asymp}} \kappa E_i - \gamma I_i^{\text{asymp}} \\
\frac{dR_i}{dt} &= +\gamma I_i + \gamma I_i^{\text{asymp}}, \qquad (1)
\end{aligned}$$

where the indices i (and j) refer to the two sub-populations, with $i = 1$ being passengers, and $i = 2$ being crew, respectively. The parameter B_{ij} describes the contact rate, sufficient to transmit infection, between individuals in sub-population i with individuals in sub-population j. Under the assumption that the probability of transmission on contact is the same for all groups, the contact rate matrix must satisfy reciprocity, implying that $N_i B_{ij} = N_j B_{ji}$ [1], where N_i is the size of sub-population i, $N_i = S_i + E_i + I_i + I_i^{\text{asymp}} + R_i$. A fraction, f_{asymp}, of exposed people move on to being asymptomatic infectious individuals, and these individuals transmit infection with a relative discount-on-transmission of σ compared to symptomatic individuals, with $0 \leq \sigma \leq 1$. In the model we neglect births and deaths.

The *next generation* matrix for this model is derived from the model *transmission matrix*, T, and the *transition matrix*, Σ [2]. The transmission matrix

for the model of Equations 1 is

$$T = \begin{bmatrix} 0 & 0 & B_{11} & B_{12}N_1/N_2 & \sigma B_{11} & \sigma B_{12}N_1/N_2 \\ 0 & 0 & B_{21}N_2/N_1 & B_{22} & \sigma B_{21}N_2/N_1 & \sigma B_{22} \\ 0 & 0 & 0 & 0 & 0 & 0 \\ 0 & 0 & 0 & 0 & 0 & 0 \\ 0 & 0 & 0 & 0 & 0 & 0 \\ 0 & 0 & 0 & 0 & 0 & 0 \end{bmatrix}. \quad (2)$$

The transition matrix for the model of Equation 1 is

$$\Sigma = \begin{bmatrix} \kappa & 0 & 0 & 0 & 0 & 0 \\ 0 & \kappa & 0 & 0 & 0 & 0 \\ -(1-f_{\text{asymp}})\kappa & 0 & \gamma & 0 & 0 & 0 \\ 0 & -(1-f_{\text{asymp}})\kappa & 0 & \gamma & 0 & 0 \\ -f_{\text{asymp}}\kappa & 0 & 0 & 0 & \gamma & 0 \\ 0 & -f_{\text{asymp}}\kappa & 0 & 0 & 0 & \gamma \end{bmatrix}. \quad (3)$$

The inverse of the transition matrix is

$$\Sigma^{-1} = \begin{bmatrix} 1/\kappa & 0 & 0 & 0 & 0 & 0 \\ 0 & 1/\kappa & 0 & 0 & 0 & 0 \\ (1-f_{\text{asymp}})/\gamma & 0 & 1/\gamma & 0 & 0 & 0 \\ 0 & (1-f_{\text{asymp}})/\gamma & 0 & 1/\gamma & 0 & 0 \\ f_{\text{asymp}}/\gamma & 0 & 0 & 0 & 1/\gamma & 0 \\ 0 & f_{\text{asymp}}/\gamma & 0 & 0 & 0 & 1/\gamma \end{bmatrix}. \quad (4)$$

The next generation matrix is defined as $K = T\Sigma^{-1}$ [2], and for the model of Equations 1 is

$$K = \begin{bmatrix} \frac{qB_{11}}{\gamma} & \frac{qB_{12}N_1}{N_2\gamma} & \frac{B_{11}}{\gamma} & \frac{B_{12}N_1}{N_2\gamma} & \frac{\sigma B_{11}}{\gamma} & \frac{\sigma B_{12}N_1}{N_2\gamma} \\ \frac{qB_{21}N_2}{N_1\gamma} & \frac{qB_{22}}{\gamma} & \frac{B_{21}N_2}{N_1\gamma} & \frac{B_{22}}{\gamma} & \frac{\sigma B_{21}N_2}{N_1\gamma} & \frac{\sigma B_{22}}{\gamma} \\ 0 & 0 & 0 & 0 & 0 & 0 \\ 0 & 0 & 0 & 0 & 0 & 0 \\ 0 & 0 & 0 & 0 & 0 & 0 \\ 0 & 0 & 0 & 0 & 0 & 0 \end{bmatrix}, \quad (5)$$

with $q = (1 - f_{\text{asymp}}) + \sigma f_{\text{asymp}}$. The reproduction number, $\mathcal{R}_0$, of this model is the largest eigenvalue of this matrix, and is

$$\mathcal{R}_0 = \frac{A + \sqrt{A^2 - 4 * C}}{2}, \tag{6}$$

with

$$A = \frac{(1 - f_{\text{asymp}}) + \sigma f_{\text{asymp}}}{\gamma} \left(B_{11} + B_{22}\right), \tag{7}$$

and

$$C = \left[\frac{(1 - f_{\text{asymp}}) + \sigma f_{\text{asymp}}}{\gamma}\right]^2 \left(B_{11}B_{22} - B_{12}B_{21}\right). \tag{8}$$

We note here in passing that the reproduction number of this model is q times the reproduction number of the model with no asymptomatic class.

1.2 Model with environmental transmission alone

Here we assume that transmission occurs via environmental transmission alone, modeled after Reference [3], with the addition of an environmental compartment, W. Again, we assume that both symptomatic and asymptomatic individuals can shed the pathogen:

$$\begin{aligned}
\frac{dS_i}{dt} &= -\eta_W W S_i \\
\frac{dE_i}{dt} &= +\eta_W W S_i - \kappa E_i \\
\frac{dI_i}{dt} &= +(1 - f_{\text{asymp}})\kappa E_i - \gamma I_i \\
\frac{dI_i^{\text{asymp}}}{dt} &= +f_{\text{asymp}}\kappa E_i - \gamma I_i^{\text{asymp}} \\
\frac{dR_i}{dt} &= +\gamma I_i + \gamma I_i^{\text{asymp}} \\
\frac{dW}{dt} &= +\alpha \sum \left(I_i + \sigma I_i^{\text{asymp}}\right) - \xi W, \qquad (9)
\end{aligned}$$

where the sub-population index, i, refers to "passengers" when $i = 1$, and "crew" when $i = 2$. The parameters α and ξ are the excretion and decay rates of the pathogen into and out of the environment, respectively, η_W is the rate at which the population contacts the environment.

Following Reference [3], we re-scale the environmental compartment, such that $W_{\text{new}} \to \frac{\xi}{\alpha N} W_{\text{old}}$. This yields

$$\begin{aligned}
\frac{dS_i}{dt} &= -\beta_W W S_i \\
\frac{dE_i}{dt} &= +\beta_W W S_i - \kappa E_i \\
\frac{dI_i}{dt} &= +(1 - f_{\text{asymp}})\kappa E_i - \gamma I_i \\
\frac{dI_i^{\text{asymp}}}{dt} &= +f_{\text{asymp}}\kappa E_i - \gamma I_i^{\text{asymp}} \\
\frac{dR_i}{dt} &= +\gamma I_i + \gamma I_i^{\text{asymp}} \\
\frac{dW}{dt} &= +\xi \left(\sum (I_i + \sigma I_i^{\text{asymp}})/N - W \right),
\end{aligned} \tag{10}$$

with $N = N_{\text{pass}} + N_{\text{crew}}$, and $\beta_W = \eta_W N\alpha/\xi$.

The transmission matrix of the model of Equations 10 is

$$T = \begin{bmatrix}
0 & 0 & 0 & 0 & 0 & 0 & N_1\beta_W \\
0 & 0 & 0 & 0 & 0 & 0 & N_2\beta_W \\
0 & 0 & 0 & 0 & 0 & 0 & 0 \\
0 & 0 & 0 & 0 & 0 & 0 & 0 \\
0 & 0 & 0 & 0 & 0 & 0 & 0 \\
0 & 0 & 0 & 0 & 0 & 0 & 0 \\
0 & 0 & 0 & 0 & 0 & 0 & 0
\end{bmatrix}. \tag{11}$$

The transition matrix of the model of Equations 10 is

$$\Sigma = \begin{bmatrix}
\kappa & 0 & 0 & 0 & 0 & 0 & 0 \\
0 & \kappa & 0 & 0 & 0 & 0 & 0 \\
-(1 - f_{\text{asymp}})\kappa & 0 & \gamma & 0 & 0 & 0 & 0 \\
0 & -(1 - f_{\text{asymp}})\kappa & 0 & \gamma & 0 & 0 & 0 \\
-f_{\text{asymp}}\kappa & 0 & 0 & 0 & \gamma & 0 & 0 \\
0 & -f_{\text{asymp}}\kappa & 0 & 0 & 0 & \gamma & 0 \\
0 & 0 & -\xi/N & -\xi/N & -\sigma\xi/N & -\sigma\xi/N & \xi
\end{bmatrix}. \tag{12}$$

The inverse of the transition matrix is

$$\Sigma^{-1} = \begin{bmatrix} 1/\kappa & 0 & 0 & 0 & 0 & 0 & 0 \\ 0 & 1/\kappa & 0 & 0 & 0 & 0 & 0 \\ (1-f_{\mathrm{asymp}})/\gamma & 0 & 1/\gamma & 0 & 0 & 0 & 0 \\ 0 & (1-f_{\mathrm{asymp}})/\gamma & 0 & 1/\gamma & 0 & 0 & 0 \\ f_{\mathrm{asymp}}/\gamma & 0 & 0 & 0 & 1/\gamma & 0 & 0 \\ 0 & f_{\mathrm{asymp}}/\gamma & 0 & 0 & 0 & 1/\gamma & 0 \\ q/N\gamma & q/N\gamma & 1/N\gamma & 1/N\gamma & \sigma/N\gamma & \sigma/N\gamma & 1/\xi \end{bmatrix} \quad (13)$$

with $q = (1 - f_{\mathrm{asymp}}) + \sigma f_{\mathrm{asymp}}$.

The next generation matrix, $K = T\Sigma^{-1}$ is

$$K = \begin{bmatrix} qf_1\frac{\beta_W}{\gamma} & qf_1\frac{\beta_W}{\gamma} & f_1\frac{\beta_W}{\gamma} & f_1\frac{\beta_W}{\gamma} & \sigma f_1\frac{\beta_W}{\gamma} & \sigma f_1\frac{\beta_W}{\gamma} & N_1\beta_W/\xi \\ qf_2\frac{\beta_W}{\gamma} & qf_2\frac{\beta_W}{\gamma} & f_2\frac{\beta_W}{\gamma} & f_2\frac{\beta_W}{\gamma} & \sigma f_2\frac{\beta_W}{\gamma} & \sigma f_2\frac{\beta_W}{\gamma} & N_2\beta_W/\xi \\ 0 & 0 & 0 & 0 & 0 & 0 & 0 \\ 0 & 0 & 0 & 0 & 0 & 0 & 0 \\ 0 & 0 & 0 & 0 & 0 & 0 & 0 \\ 0 & 0 & 0 & 0 & 0 & 0 & 0 \\ 0 & 0 & 0 & 0 & 0 & 0 & 0 \end{bmatrix}, \quad (14)$$

where f_i is the fraction of the population in sub-population i. The largest eigenvalue of this matrix is $\mathcal{R}_0 = q\frac{\beta_W}{\gamma}$. We find that the reproduction number in this case does not depend on the population sub-structure.

Since $\beta_W = N\eta_W\alpha/\xi$, we can re-cast the expression for the reproduction number as $\mathcal{R}_0 = q\frac{N\eta_W\alpha}{\gamma\xi}$, which makes somewhat more explicit the dependence of the reproduction number on the environmental contact rate, excretion rate, and decay rate.

1.3 Model with both direct and environmental transmission

Here we assume that transmission occurs via both environmental transmission and direct transmission, with environmental transmission modeled after Reference [3], with the addition of an environmental compartment, W. We again

include both symptomatic and asymptomatic infectious classes:

$$\begin{aligned}
\frac{dS_i}{dt} &= -\eta_W W S_i - S_i \sum_j B_{ij} I_j/N_j - \sigma S_i \sum_j B_{ij} I_j^{\text{asymp}}/N_j \\
\frac{dE_i}{dt} &= +\eta_W W S_i + S_i \sum_j B_{ij} I_j/N_j + \sigma S_i \sum_j B_{ij} I_j^{\text{asymp}}/N_j - \kappa E_i \\
\frac{dI_i}{dt} &= +(1 - f_{\text{asymp}})\kappa E_i - \gamma I_i \\
\frac{dI_i^{\text{asymp}}}{dt} &= +f_{\text{asymp}} \kappa E_i - \gamma I_i^{\text{asymp}} \\
\frac{dR_i}{dt} &= +\gamma I_i + \gamma I_i^{\text{asymp}} \\
\frac{dW}{dt} &= +\alpha \sum I_i + \sigma\alpha \sum I_i^{\text{asymp}} - \xi W,
\end{aligned} \tag{15}$$

where the sub-population index, i, refers to "passengers" when $i = 1$, and "crew" when $i = 2$. The parameter B_{ij} is the contact rate, sufficient to transmit infection, between individuals in sub-population i, and those in sub-population j. The parameter η_W is the rate at which the population contacts the environment, and α and ξ are the excretion and decay rates of the pathogen into, and out of, the environment, respectively. The population size is $N = N_{\text{pass}} + N_{\text{crew}}$.

Following Reference [3], we re-scale the environmental compartment of Equations 15, such that $W_{\text{new}} \to \frac{\xi}{\alpha N} W_{\text{old}}$. This yields

$$\begin{aligned}
\frac{dS_i}{dt} &= -\beta_W W S_i - S_i \sum_j B_{ij} I_j/N_j - \sigma S_i \sum_j B_{ij} I_j^{\text{asymp}}/N_j \\
\frac{dE_i}{dt} &= +\beta_W W S_i + S_i \sum_j B_{ij} I_j/N_j + \sigma S_i \sum_j B_{ij} I_j^{\text{asymp}}/N_j - \kappa E_i \\
\frac{dI_i}{dt} &= +(1 - f_{\text{asymp}})\kappa E_i - \gamma I_i \\
\frac{dI_i^{\text{asymp}}}{dt} &= +f_{\text{asymp}} \kappa E_i - \gamma I_i^{\text{asymp}} \\
\frac{dR_i}{dt} &= +\gamma I_i + \gamma I_i^{\text{asymp}} \\
\frac{dW}{dt} &= +\xi \left(\sum (I_i + \sigma I_i^{\text{asymp}})/N - W \right),
\end{aligned} \tag{16}$$

with scaled environmental transmission rate $\beta_W = \eta_W N \alpha/\xi$.

The transmission matrix of Equations 16 is

$$T = \begin{bmatrix} 0 & 0 & B_{11} & B_{12}N_1/N_2 & \sigma B_{11} & \sigma B_{12}N_1/N_2 & N_1\beta_W \\ 0 & 0 & B_{21}N_2/N_1 & B_{22} & \sigma B_{21}N_2/N_1 & \sigma B_{22} & N_2\beta_W \\ 0 & 0 & 0 & 0 & 0 & 0 & 0 \\ 0 & 0 & 0 & 0 & 0 & 0 & 0 \\ 0 & 0 & 0 & 0 & 0 & 0 & 0 \\ 0 & 0 & 0 & 0 & 0 & 0 & 0 \\ 0 & 0 & 0 & 0 & 0 & 0 & 0 \end{bmatrix} \tag{17}$$

The transition matrix is

$$\Sigma = \begin{bmatrix} \kappa & 0 & 0 & 0 & 0 & 0 & 0 \\ 0 & \kappa & 0 & 0 & 0 & 0 & 0 \\ -(1-f_{\text{asymp}})\kappa & 0 & \gamma & 0 & 0 & 0 & 0 \\ 0 & -(1-f_{\text{asymp}})\kappa & 0 & \gamma & 0 & 0 & 0 \\ -f_{\text{asymp}}\kappa & 0 & 0 & 0 & \gamma & 0 & 0 \\ 0 & -f_{\text{asymp}}\kappa & 0 & 0 & 0 & \gamma & 0 \\ 0 & 0 & -\xi/N & -\xi/N & -\sigma\xi/N & -\sigma\xi/N & \xi \end{bmatrix}. \tag{18}$$

The inverse of the transition matrix is

$$\Sigma^{-1} = \begin{bmatrix} 1/\kappa & 0 & 0 & 0 & 0 & 0 & 0 \\ 0 & 1/\kappa & 0 & 0 & 0 & 0 & 0 \\ (1-f_{\text{asymp}})/\gamma & 0 & 1/\gamma & 0 & 0 & 0 & 0 \\ 0 & (1-f_{\text{asymp}})/\gamma & 0 & 1/\gamma & 0 & 0 & 0 \\ f_{\text{asymp}}/\gamma & 0 & 0 & 0 & 1/\gamma & 0 & 0 \\ 0 & f_{\text{asymp}}/\gamma & 0 & 0 & 0 & 1/\gamma & 0 \\ q/N\gamma & q/N\gamma & 1/N\gamma & 1/N\gamma & \sigma/N\gamma & \sigma/N\gamma & 1/\xi \end{bmatrix}, \tag{19}$$

with $q = (1 - f_{\text{asymp}}) + \sigma f_{\text{asymp}}$.

The next generation matrix, $K = T\Sigma^{-1}$ is

$$K = \begin{bmatrix} qr & qs & r & s & \sigma r & \sigma s & N_1\beta_W/\xi \\ qt & qu & t & u & \sigma t & \sigma u & N_2\beta_W/\xi \\ 0 & 0 & 0 & 0 & 0 & 0 & 0 \\ 0 & 0 & 0 & 0 & 0 & 0 & 0 \\ 0 & 0 & 0 & 0 & 0 & 0 & 0 \\ 0 & 0 & 0 & 0 & 0 & 0 & 0 \\ 0 & 0 & 0 & 0 & 0 & 0 & 0 \end{bmatrix}, \tag{20}$$

with

$$r = \frac{B_{11}}{\gamma} + f_1\frac{\beta_W}{\gamma} \tag{21}$$
$$s = \frac{B_{12}N_1}{N_2\gamma} + f_1\frac{\beta_W}{\gamma} \tag{22}$$
$$t = \frac{B_{21}N_2}{N_1\gamma} + f_2\frac{\beta_W}{\gamma} \tag{23}$$
$$u = \frac{B_{22}}{\gamma} + f_2\frac{\beta_W}{\gamma}, \tag{24}$$

where $f_1 = N_1/N$, and $f_2 = N_2/N$.

The largest eigenvalue of this matrix (and reproduction number of the model of Equations 16) is the largest solution to

$$\lambda^2 - q(r+u)\lambda + q^2(ru - st) = 0. \tag{25}$$

Which yields

$$\mathcal{R}_0 = q\frac{(r+u) + \sqrt{(r+u)^2 - 4*ru + 4*ts}}{2} \tag{26}$$
$$= q\frac{(r+u) + \sqrt{(r-u)^2 + 4*ts}}{2} \tag{27}$$
$$= q\frac{B_{11} + B_{22} + \beta_W + A}{2\gamma}, \tag{28}$$

where

$$A = \sqrt{(B_{11} - B_{22} + (f_1 - f_2)\beta_W)^2 + 4*f_1*f_2(B_{12}/f_2 + \beta_W)(B_{21}/f_1 + \beta_W)}.$$

1.4 Model with both direct and environmental transmission, and isolation

Here we assume, as in the previous section, that transmission occurs via both environmental transmission and direct transmission, and that a fraction, $f_{\rm isol}$ of symptomatic passengers are immediately, and completely effectively, isolated to compartment H upon becoming infectious and symptomatic:

$$\begin{aligned}
\frac{dS_i}{dt} &= -\eta_W W S_i - S_i \sum_j B_{ij} I_j/N_j - \sigma S_i \sum_j B_{ij} I_j^{\rm asymp}/N_j \\
\frac{dE_i}{dt} &= +\eta_W W S_i + S_i \sum_j B_{ij} I_j/N_j + \sigma S_i \sum_j B_{ij} I_j^{\rm asymp}/N_j - \kappa E_i \\
\frac{dI_i}{dt} &= +(1-f_{\rm isol})(1-f_{\rm asymp})\kappa E_i - \gamma I_i \\
\frac{dI_i^{\rm asymp}}{dt} &= +f_{\rm asymp}\kappa E_i - \gamma I_i^{\rm asymp} \\
\frac{dH_i}{dt} &= f_{\rm isol}(1-f_{\rm asymp})\kappa E_i - \gamma H_i \\
\frac{dR_i}{dt} &= +\gamma I_i + \gamma I_i^{\rm asymp} + \gamma H_i \\
\frac{dW}{dt} &= +\alpha \sum I_i + \sigma\alpha \sum I_i^{\rm asymp} - \xi W, \qquad (29)
\end{aligned}$$

with population size $N = S_{\rm crew} + E_{\rm crew} + I_{\rm crew} + I_{\rm crew}^{\rm asymp} + H_{\rm crew} + R_{\rm crew} + S_{\rm pass} + E_{\rm pass} + I_{\rm pass} + I_{\rm pass}^{\rm asymp} + H_{\rm pass} + R_{\rm pass}$.

Because the isolated class cannot contribute to the chain of infection, the reproduction number calculation for this model follows that of the previous section, except all instances of $(1-f_{\rm asymp})$ in the transition matrix are replaced by $(1-f_{\rm isol})(1-f_{\rm asymp})$.

The reproduction number of the model of Equations 29 is the largest solution to

$$\lambda^2 - z(r+u)\lambda + z^2(ru - st) = 0, \qquad (30)$$

with $z = (1 - f_{\text{isol}})(1 - f_{\text{asymp}}) + \sigma f_{\text{asymp}}$. Which yields

$$\begin{aligned} \mathcal{R}_0 &= z\frac{(r+u) + \sqrt{(r+u)^2 - 4*ru + 4*ts}}{2} && (31) \\ &= z\frac{(r+u) + \sqrt{(r-u)^2 + 4*ts}}{2} && (32) \\ &= z\frac{B_{11} + B_{22} + \beta_W + A}{2\gamma}, && (33) \end{aligned}$$

where

$$A = \sqrt{(B_{11} - B_{22} + (f_1 - f_2)\beta_W)^2 + 4*f_1*f_2(B_{12}/f_2 + \beta_W)(B_{21}/f_1 + \beta_W)}.$$

1.5 Pre-existing immunity, and the effective reproduction number

If some fraction, f_{susc} of the population is susceptible to being infected with a pathogen, and thus $(1 - f_{\text{susc}})$ is immune, the effective reproduction number at the beginning of the outbreak is [4]

$$\mathcal{R}_{\text{eff}} = f_{\text{susc}}\mathcal{R}_0. \tag{34}$$

Pre-existing immunity can come either from previous infection, or vaccination.

In this analysis we assume that an equal fraction of crew and passengers are susceptible.

References

[1] Wallinga J, Teunis P, Kretzschmar M. Using data on social contacts to estimate age-specific transmission parameters for respiratory-spread infectious agents. American journal of epidemiology. 2006;164(10):936–944.

[2] Diekmann O, Heesterbeek J, Roberts M. The construction of next-generation matrices for compartmental epidemic models. Journal of the Royal Society Interface. 2009;p. rsif20090386.

[3] Tien JH, Earn DJ. Multiple transmission pathways and disease dynamics in a waterborne pathogen model. Bulletin of mathematical biology. 2010;72(6):1506–1533.

[4] Hethcote HW. The mathematics of infectious diseases. SIAM review. 2000;42(4):599–653.
